# Prevalence and types of high-risk human papillomaviruses in head and neck cancers from Bangladesh

**DOI:** 10.1186/s12885-017-3789-0

**Published:** 2017-11-25

**Authors:** Mushfiq H. Shaikh, Aminul I. Khan, Anwar Sadat, Ahmed H. Chowdhury, Shahed A. Jinnah, Vinod Gopalan, Alfred K. Lam, Daniel T. W. Clarke, Nigel A. J. McMillan, Newell W. Johnson

**Affiliations:** 1 School of Dentistry and Oral Health, Griffith University, Gold Coast, QLD 4222 Australia; 2 School of Medical Science, Griffith University, Gold Coast, QLD 4222 Australia; 3 Understanding Chronic Conditions Program, Menzies Health Institute Queensland, Gold Coast, QLD 4222 Australia; 4 Department of Pathology, National Medical College and Hospital, Dhaka, 1100 Bangladesh; 5 Department of Oral and Maxillo-facial Surgery, Dhaka Dental College & Hospital, Dhaka, 1216 Bangladesh; 6 Department of Neuro-medicine, Dhaka Medical College and Hospital, Dhaka, 1000 Bangladesh; 7 Department of Pathology, Dhaka Medical College and Hospital, Dhaka, 1000 Bangladesh; 8 School of Medicine, Griffith University, Gold Coast, QLD 4222 Australia; 9 Dental Institute, King’s College London, London, UK; 10 Menzies Health Institute Queensland and School of Dentistry and Oral Health, Griffith University, Building G40, Room 9.16, Gold Coast Campus, Gold Coast, QLD 4222 Australia

**Keywords:** Head and neck squamous cell carcinoma (HNSCC), Human papillomavirus (HPV), Nested polymerase chain reaction (PCR), Immunohistochemistry (IHC), South Asia, Bangladesh

## Abstract

**Background:**

There is a dramatic rise in the incidence of Human papillomavirus (HPV) – associated head and neck squamous cell carcinoma (HNSCC) in the world, with considerable variation by geography, gender and ethnicity. Little is known about the situation in Bangladesh, where tobacco- and areca nut-related head and neck cancers (HNCs) are the most common cancers in men. We aimed to determine the prevalence of HPV in HNSCC in Bangladesh and to explore the possible value of cell cycle markers in clinical diagnostic settings.

**Methods:**

One hundred and ninety six archival HNSCC tissue samples were analysed for the presence of HPV DNA. The DNA quality was assured, and then amplified using a nested PCR approach. The typing of HPV was performed by automated DNA sequencing. Cellular markers p53, Cyclin D1 and pRb were tested on all samples by immunohistochemistry (IHC), as well as p16 as a putative surrogate for the detection of HPV.

**Results:**

HPV DNA was detected in 36/174 (~21%) samples: 36% of cancers from the oropharynx; 31% of oral cancers, and 22% from the larynx. HPV-16 was most common, being present in 33 samples, followed by HPV-33 (2 samples) and HPV-31 (1 sample). Twenty-eight out of 174 samples were positive for p16, predominantly in HPV-positive tissues (*p* < 0.001). No statistically significant association was observed between the cellular markers and HPV DNA positive cases. However, p16 positivity had excellent predictive value for the presence of HPV by PCR.

**Conclusion:**

There is a significant burden of HPV-associated HNSCC in Bangladesh, particularly in the oropharynx but also in oral and laryngeal cancers. Whilst a combination of PCR-based DNA detection and p16 IHC is useful, the latter has excellent specificity, acceptable sensitivity and good predictive value for carriage of HPV in this population and should be used for prognostic evaluation and treatment planning of all HNSCC patients in South Asia, as in the Western world.

## Background

Head and neck cancer (HNC) is a major health problem worldwide, with an annual incidence of approximately 600,000 cases and close to 300,000 deaths, mostly in less developed countries (GLOBOCAN 2012) [1]. Whilst most – 80-90% in some countries - are squamous cell carcinomas and variants thereof, neoplasms in this region are diverse at clinical and biological levels, which make them difficult to manage. Although tobacco, areca nut and alcohol are the major risk factors for HNSCC, infection with high-risk types of Human papillomavirus (HPV) has been shown to be strongly associated with a significant proportion of cases [2]. This association varies by anatomical site/subsite, with a predilection for mucosa associated with the lymphoid aggregations of Waldeyer’s ring, and are thus seen in the oropharynx, especially base of tongue and palatine tonsils, compared to the oral cavity, larynx and hypo-pharynx [3]. Recently, the International Agency for Research on Cancer (IARC) has acknowledged HPV as an aetiological factor for oropharyngeal squamous cell carcinoma (OPSCC) [4, 5]. HPV-associated HNSCC represents a distinct entity with increase in incidence over the last three decades, mostly in developed countries and commonly affecting young adult males who tend to be non-smokers, non- or light- drinkers and many have relatively high socioeconomic status [6]. It is suggested that this is related to changing sexual behaviour, with an increase in oral-genital contact [7], sexual debut at early age and a high number of lifetime sex partners [8]. Because HPV-related HNC patients have significantly better treatment response and 3-year overall survival rates (82.4% vs 57.1%) irrespective of age, gender or tumour stage [9, 10], knowledge of HPV status is mandatory in most tumour boards for the planning of treatment.

Approximately one-third of the total HNC cases in the world have been shown to be associated with high-risk HPV infection, but wide geographic variation exists [9, 11].

The prevalence of HPV in HNC, especially, OPSCC is much higher in North America (~70%) and Europe (~50%) compared to rest of the world [12]. An increasing trend is noticed in Australia, where HPV-positive OPSCC rose from 20% to 63% of cases over the last two decades [13]. South Asia (including the Indian subcontinent) has the highest incidence rates and disease burden of HNSCC in the world with approximately 200,000 new cases each year and more than 100,000 deaths (GLOBOCAN 2012) [1, 11]. However, relevant studies in South Asian populations are few and inconsistent. HNSCC is *the leading* cancer in males and 3rd most common in females in India [14]. Bangladesh shares similar cultural & social norms as India. Likewise, HNSCC is also the most common cancer in males in Bangladesh, surpassing lung cancer [15]. As extensive use of tobacco (in smoking and smokeless forms) and chewing of areca nut dominate the risks for head and neck cancer across South Asia, less attention has been given to the role of HPV. There are no comprehensive data from Bangladesh. Because of the high burden of this disease in South and South East Asia it is essential to have accurate data on the role of HPV across the region.

The primary objective of our study was to investigate the prevalence of high risk HPV in HNSCC in a Bangladeshi cohort of patients assembling tumours from different sites of the head and neck region. We also determined the concordance between commonly used HPV detection methods, namely polymerase chain reaction (detects the presence of HPV DNA in tumour tissue) and p16 immunohistochemistry (IHC), a commonly used surrogate marker for HPV-associated cancers. Increased p16 expression is a direct consequence of E7 (HPV oncoprotein) -induced retinoblastoma (pRb) protein inactivation (no/low expression) [16], and Cyclin D1 protein expression is dependent on intact pRb expression [17]. Thus, analyzing the expression of both pRb and Cyclin D1 could provide useful prognostic information about the biological activity of HPV in HNSCC. Further, HPV-positive HNSCC is associated with low level of p53 expression due to the suppressing action of another viral oncoprotein, HPV E6 [18]. Based on these considerations, here we address the correlation of the potential prognostic markers p16, p53, pRb and Cyclin D1 with HPV status. Our data provide insights into the relative burden and aetiology of HNCs in Bangladesh, likely generalizable to South Asia as a whole.

## Methods

### Study population and data collection

A total of 196 de-identified HNSCC cases were included. Patients were from Dhaka Medical College Hospital (DMCH), A.I. Khan Laboratory and Millennium Dental Clinic in the city. All were over 20 years of age, clinically and pathologically diagnosed with head and neck cancer between December 2014 and May 2016. The cancers were classified into different subsites of the head and neck following the ICD-10 classification. The borders of tongue (C02.1), gingiva (C03), floor of the mouth (C04), hard palate (C05.0), buccal mucosa (C06.0), vestibular fold (C06.1) and retromolar area (C06.2) were grouped under ‘oral cavity’ (C02-C06), while the base of the tongue (C01), soft palate (C05.1), tonsils (C09.9), vallecula (C10.0) and pharyngeal walls (C10.2 & C10.3) were congregated under ‘oropharynx’ (C01, C09 & C10). Further, pyriform fossa (C12) and cricoid region (C13) were classified under ‘hypopharynx’ (C12, C13) and the supraglottic (C32.1), vocal cord (C32.0), epiglottic (C32.1) and aryepiglottic fold (C32.1) were grouped under ‘larynx’ (C32). Cancers of the salivary glands and nasopharynx were excluded. Diagnostic biopsy specimens were preserved as formalin fixed paraffin embedded (FFPE) blocks in the Department of Pathology, DMCH and in A. I. Khan Pathology Laboratory, Dhanmondi, and Dhaka. Clinico-pathological data of tumour sites, tumour differentiation, and demography of patients were retrieved from the pathological records.

The study was approved by the Griffith University Human Research Ethics Committee in Australia (GU Ref No: DOH/13/14/HREC) and DMCH Human Ethics Committee (Memo No. DMC/ECC/2016/32) in Bangladesh.

### HPV DNA detection & type determination

#### DNA isolation and testing of sample integrity

The FFPE blocks were sectioned whilst maintaining utmost precautions to avoid inter-block contamination of DNA. This was achieved by pre-chilling and moistening each block in a separate ice container before sectioning, thorough cleaning of the microtome, single use of brush and forceps, changing gloves in between each block, changing the water bath for each block and using a new blade for each block. Genomic DNA was extracted from a 10 um thick section, using GeneRead FFPE kit (Qiagen, Germany). A section from a blank paraffin block was cut and processed along with the sections from the cases to check for any contamination. DNA concentrations were determined with a NanoDrop 2000 spectrophotometer (Thermo Scientific, Waltham, MA). DNA integrity was assessed for 150 bp fragments of the Beta-actin housekeeping gene by PCR using the GoTaq Green PCR kit (Promega, Madison, WI). Nuclease free water was used as negative control, while genomic DNA from the HPV16 positive head and neck cancer cell line, UDSCC-2 (kindly provided by Dr. Silke Schwarz and Prof. Thomas Hoffman, University of Ulm, Germany) and HPV-negative head and neck cancer cell line, SCC25 were used as a positive control and a negative tissue control, respectively, in the first PCR run. From the 2nd PCR run onwards, samples that had shown positivity and negativity in the first PCR run were used as positive and negative controls, respectively.

### Detection of HPV DNA by PCR

Nested PCR, consisting of two sets of degenerative/consensus primer pairs, MY09/11 and GP5+/GP6+ (Sigma-Aldrich, St. Louis, MO, USA) were used [19–21]. A gradient PCR was performed to optimise the annealing temperature for each primer set. The HPV *L1* gene was amplified using primers MY09/11 in the first round, followed by GP5+/GP6+ in the second round. The PCR reaction mix contained forward and reversed primers (0.5 μM of each), 1 × PCR buffer (containing 1.5 mM MgCl_2_) (Phusion High-Fidelity 5 × PCR Buffer, New England Biolabs, MA, USA), 200 μM of dNTPs (10 mM dNTP Mix, New England Biolabs, MA, USA), 1.0 unit of Phusion DNA polymerase (Taq polymerase 1unit/50 μl, New England Biolabs, MA, USA) and nuclease-free water, up to a final volume of 20 μl. Positive and negative reaction controls were included in each PCR run. DNA amplification was carried out in an automated thermal cycler (Takara Bio Inc., Japan). Reactions were brought to 98 °C for 30 s (initial denaturation), followed by forty cycles consisting of a denaturing step for 10 s at 98 °C, an annealing step for 30 s at 55.5 °C (MY09/11 in first round) or 55 °C (GP5+/GP6+ second round), and an extension step for 20 s at 72 °C. A final extension step at 72 °C was carried out for 5 min. A total of 2 μl of the first-round PCR product was used in the second round of amplifications.

The PCR products of the samples from both rounds were electrophoresed in 2% agarose gel prepared with 1× TAE (Tris-acetate-EDTA) buffer (DNA Agar, Marine Bio Products Inc., Quincy, MA, USA), stained with 0.5 g/mol of ethidium bromide (Merck, KGaA, Darmstadt, Germany) and visualized under ultraviolet light using the Chemi-Doc machine (BioRad, USA). The size of the amplified product was determined by comparing with a reference molecular weight DNA marker, (Quick Load, 100 bp DNA Ladder, New England Biolab, MA, USA). Any sample that showed a positive band in the gel for both first (band size 450 bp) and second (band size 150 bp) round of PCR was taken as an HPV-positive sample and purified for sequencing.

### Sequencing for HPV type

A PCR DNA purification kit (Qiagen, Germany) was used to purify the PCR product of the HPV-DNA positive samples detected in gel electrophoresis. These were submitted to the Australian Genome Research Facility (AGRF) for automated sequencing. Sequences were compared with available HPV genome sequences in Genebank using the NCBI (National Center for Biotechnology Information) Blast programme.

### Histological diagnosis

Adjacent haematoxylin and eosin (H&E) stained sections were used to confirm diagnoses and to grade. Grades 1, 2 and 3 were referred to as well, moderately and poorly differentiated, respectively [22].

### Immunohistochemical analysis

Immunohistochemistry (IHC) for p16, p53, pRb and Cyclin D1 was performed in the laboratories of Menzies Health Institute Queensland (MHIQ) and/or Gold Coast University Hospital, using DAKO histology kits (EnVision FLEX Mini Kit, High pH) (DAKO, Agilent, Santa Clara, CA), some manually, others in an Intellipath (Biocare Medical, Concord, CA) autostainer. Briefly, from each FFPE block, a 4um thick section was cut and affixed to Menzel-Gläser super-frost plus slides (Thermo Fisher Scientific, Waltham, MA) and air-dried at 37 °C for 48 h. Slides were preheated at 60 °C, followed by de-waxing and re-hydration using xylene, ethanol and water. Antigen retrieval was performed with the DAKO EnVision Kit (DAKO, Agilent, Santa Clara, CA) followed by wash with Tris-Buffer Saline (TBS) mixed with 0.1% Tween 20. Staining with primary antibodies was carried out according to the protocol recommended by DAKO, using an Intellipath autostainer, where staining steps and incubation times were programmed according to the DAKO EnVision FLEX Mini Kit protocol. A similar procedure was followed for manual staining. The primary antibodies mouse anti-p16^INK4a^ (#2D9A12; DAKO, Agilent, Santa Clara, CA), mouse anti-human p53 (#DO-7; DAKO, Agilent, Santa Clara, CA), rabbit anti-human Cyclin D1 (#EP12, DAKO, Agilent, Santa Clara, CA), and rabbit anti-human pRB (#9308; Cell Signaling, Danvers, MA) were optimized for antibody concentration and incubation time according to their respective company protocols. Positive and negative IHC controls were used in every run. A tonsillar SCC with high p16 expression was taken as positive control for p16 IHC; for p53 a positive colon cancer; for Cyclin D1 and pRb, a known positive tonsillar carcinoma.

**Fig. 1:**
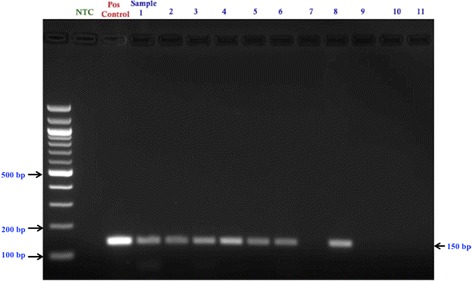
Gel electrophoresis of the 2nd round Nested PCR of 11 samples. The product size is 150 base pairs. Seven of 11 samples are showing positive for HPV DNA

IHC slides were scored independently by three head and neck pathologists (AL, MS & VG). Discordant cases were few and agreed by discussion. Cases with moderate to strong staining of all neoplastic areas were recorded as positive, while sections with weak and focal staining were regarded as negative. To be regarded as p16 positive, sections had to show both nuclear and cytoplasmic staining in 50% or more of neoplastic cells [23, 24]. For p53, Cyclin D1 and pRb proteins, in which reaction product is present in nuclei only, slides were scored dichotomously, negative being <10% of cells staining, positive being >10% [25–27]. Typical staining patterns of each cellular marker are presented in Fig. 1 (a–d).

### Statistical analysis

This was carried out using SPSS version 22 (IBM Corporation, Armonk, NY). To compare the characteristics of HPV-positive and -negative patients, χ^2^ or Fischer’s exact tests were used. However, to determine the mean age differences between HPV-positive and -negative groups, an independent t-test were used. Spearman’s rank coefficient was used to analyse possible correlations among and between p16, p53, pRb and cyclin D1 expression levels. All analyses were two-sided and *p*-values below 0.05 were regarded as significant. In addition, the predictive values of p16 and PCR-determined HPV status were examined by standard 2X2 table analyses, and the sensitivity and specificity calculated [28, 29].

## Results

### Patient demography and histology

A total of 174 of the 196 blocks were analysed: 22 did not contain PCR-amplifiable DNA as determined by β-actin. The mean age of all patients was 56.6 years (Table 1): 138 (~80%) were men and 36 (~20%) women. Primary tumour sites, as given in pathology records, were: oral cavity 55 (31.6%), oropharynx 35 (20.1%), larynx 64 (36.8%) and hypopharynx 20 (11.5%). The majority, 98 (56.3%), were moderately differentiated; followed by well- 66 (37.9%) and poorly differentiated 10 (5.7%) (Table 1).

**Table 1:** Demographic and clinical characteristics of patients

Characteristics	All patients (*n* = 174) (%)	HPV DNA + ve (*n* = 36) (%)	HPV DNA-ve (*n* = 138)(%)	X^2^ (Chi-square)	*p*-value
Mean Age	56.6	54.2	57.2		0.143
Age groups (years)					
20–59	94 (54.1)	26 (72.2)	68 (49.3)	6.053*	0.014
60 and above	80 (45.9)	10 (27.8)	70 (50.7)		
Gender					
Male	138 (79.3)	30 (83.3)	108 (78.3)	0.448	0.503
Female	36 (20.7)	6 (16.7)	30 (21.7)		
Primary Tumour sites					
Oral Cavity (C02-C06)	55 (31.6)	11 (30.6)	44 (31.9)	8.412*	0.038
Oropharynx (C01, C09 & C10)	35 (20.1)	13 (36.1)	22 (15.9)		
Larynx (C32)	64 (36.8)	8 (22.2)	56 (40.6)		
Hypopharynx (C13)	20 (11.5)	4 (11.1)	16 (11.6)		
Histopathological Grading					
Well differentiated	48 (27.6)	4 (11.1)	44 (31.9)	8.947*	0.011
Moderately differentiated	92 (52.9)	20 (55.6)	72 (52.2)		
Poorly differentiated	34 (19.5)	12 (33.3)	22 (15.9)		
Immunohistochemistry (IHC) Analysis					
p16 expression					
Positive	28 (16.1)	26 (72.2)	2 (1.4)	105.914*	0.0001
Negative	146 (83.9)	10 (27.8)	136 (98.6)		
p53 expression					
Positive	86 (49.4)	23 (63.9)	63 (45.7)	3.799	0.051
Negative	88 (51.6)	13 (36.1)	75 (54.3)		
Cyclin D1 expression					
Positive	62 (35.6)	13 (36.1)	49 (35.5)	0.05	0.946
Negative	112 (64.4)	23 (63.9)	89 (64.5)		
pRb expression					
Positive	66 (37.9)	15 (41.7)	51 (37.0)	0.269	0.604
Negative	108 (62.1)	21 (58.3)	87 (63.0)		

*indicates statistical significance

### Presence of HPV DNA and HPV type

Overall, 36/174 (~21%) of blocks were positive for HPV DNA. The HPV prevalence was significantly higher for tumours in the oropharynx, 13/35 (37.1%), followed by oral cavity, 11/55 (20.0%) and larynx, 8/64 (12.5%) and hypopharynx (20.0%). Sequencing showed that HPV-16 was most common, 33/36 (~92%) of the HPV-positive tumours, followed by HPV-31 (2 cases) (~6%) and HPV-33 (1 case) (~3%).

**Fig. 2:**
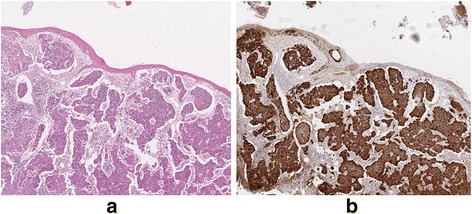
**a** Poorly differentiated HPV-associated SCC of tonsil (H&E), in this area beneath an intact surface, (**b**) Intense staining of nuclei and cytoplasm for p16 in the same tumour (20X magnification of original image)

HPV-positive patients were younger (mean ~54 years) than HPV-negative cases (mean ~ 57 years) and HPV prevalence was inversely correlated with age, (*p* = 0.014). A higher proportion of cases in men were HPV-positive (30/138, 21.7% cf. 6/36, 16.6% for women) but this was not statistically significant (Table 1). Among the HPV-positive cases, a significantly higher proportion fell into the moderately differentiated group (*p* = 0.011) (Table 1) (Fig. 2).

### Correlation between presence of HPV DNA and p16 expression

Only 28 of 174 samples (16.1%) exhibited p16 overexpression, mostly in HPV positive cases (*p* < 0.0001) (Table 2). Of the 36 cases positive for HPV DNA, 26 (72%) showed p16 overexpression (true positive), whereas 138 (94%) of the 146 cases were negative for both HPV DNA and p16 (true negative). From the oropharynx, 13 cases showed HPV-positivity, 11 of which were also positive for p16. Similarly, from the oral cavity, 11 cases were HPV-positive but only 6 cases showed p16 positivity. From the larynx, 8 cases were HPV positive, 6 of which were also p16 positive. From the hypopharynx, 4 cases were HPV positive, 3 of which were also p16 positive. Taking PCR data as the standard, p16 as a surrogate marker for presence of HPV showed excellent specificity of almost 99%, an acceptable sensitivity of 72%, with both Positive and Negative Predictive Values of 93% (Table 3).

**Table 2:** Concordance between HPV positive nested PCR and p16 IHC results

	HPV DNA detection by PCR		
P16 by IHC	HPV positive	HPV negative	Total
Positive	26 (14.9%)	2 (1.2%)	28 (16.1%)
Negative	10 (5.7%)	136 (78.2%)	146 (83.9%)
Total	36	138	174

**Table 3:** Sensitivity and specificity between HPV DNA detection by PCR (“positive result defined as disease for statistical testing”) and p16 by IHC

Point estimate		Confidence limits (95% CI)
Types	Value	Lower, upper
Sensitivity	72.22%	54.81% to 85.80%
Specificity	98.55%	94.86% to 99.82%
Positive Likelihood Ratio	49.83	12.40 to 200.21
Negative Likelihood Ratio	0.28	0.17 to 0.48
“Disease” prevalence	20.69% (^a^)	14.93% to 27.47%
Positive Predictive Value	92.86% (^a^)	76.50% to 99.12%
Negative Predictive Value	93.15% (^a^)	87.76% to 96.67%

^a^Does not reflect real prevalence of the “disease”

#### *Expression of cell cycle proteins:* (Fig. 3)

Strong expression of p53 was seen in overall half of cases, 86/174 (~49%), a higher proportion in the HPV-positive cases 23/36 (63.9%) compared to HPV negative cases 63/138 (45.7%). The positive cases for Cyclin D1 and pRb were 62/174 (35.6%) and 66/174 (37.9%) respectively, predominantly in HPV-negative cases: 49/62 (79%) and 51/66 (77.3%), respectively. However, none of these differences were statistically significant (Table 1).

**Fig. 3:**
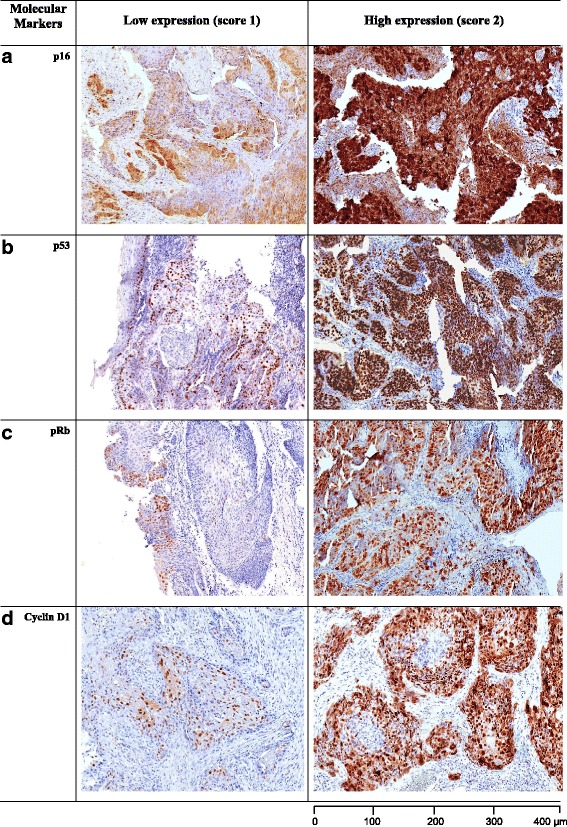
**a** Typical p16 staining of both nucleus and cytoplasm of tumour cells (20X magnification). **b** Nuclear staining pattern of tumour cells by p53 antibody (20X magnification). **c** Nuclear staining pattern of pRb (20X magnification). **d** Low to strong Cyclin D1 staining of nuclei (20X magnification)

Correlations between over-expressions of cell cycle proteins and presence of high-risk HPV infection are presented in Table 4. There was a substantial and highly significant agreement between HPV and p16 status (*p* < 0.01) and slight agreement between HPV status and p53 expression (r value 0.132). There was no agreement between HPV status and pRb expression, and disagreement between HPV status and Cyclin D1 expression (r value – 0.005). However, there was no significant correlation between the HPV status and expressions of p53, pRb and Cyclin D1 markers (Table 4).

**Table 4:** Agreement between HPV status and cell-cycle proteins in HNSCC tumours

	HPV	p16	p53	Cyclin D1	pRb
HPV	1.00				
p16	0.766**	1.00			
p53	0.132	0.224**	1.00		
Cyclin D1	- 0.005	0.01	0.177*	1.00	
pRb	- 0.001	0.077	0.222**	0.160*	1.00

**p < 0.01, **correlation is significant at 0.01 level (2-tailed)

*p < 0.05, *correlation is significant at 0.05 level (2-tailed)

Among the cell cycle proteins, a significant but weak agreement (r value 0.224) was observed between p16 and p53 expression (*p* < 0.01). However, no significant interrelationship has been identified among p16, Cyclin D1 and pRb expression. There were significant correlations between p53 & Cyclin D1 (*p* < 0.05), and between p53 & pRb (*p* < 0.01) but at weak to moderate levels (r values 0.170 and 0.216, respectively) (Table 4). A significant association is also seen between Cyclin D1 and pRb proteins but at a weak level.

## Discussion

Epidemiological, clinical & molecular studies indicate that high-risk HPV plays a pivotal role in the aetiopathogenesis of some HNSCCs. It is well documented that HPV-associated HNSCC are mostly seen in the oropharyngeal region due to the presence of lymphoid tissue, which makes it vulnerable to HPV infection [3]. An increasing trend of HPV-associated HNSCC, especially oropharyngeal cancer, is seen in developed countries, where tobacco and alcohol related HNSCC cases are decreasing [30]. Similar trends are not yet clear in South Asia, South East Asia or East Asia, because there are so far few data from these regions. Such studies as have been published have small sample sizes and have examined mostly cancers of the oral cavity, possibly because this is the dominant site for HNSCC, most of which are tobacco, areca nut and alcohol related [11].

Bangladesh, being the 3rd most populous country in South Asia and 8th in the world (~160 million), has the highest HNSCC incidence in the region, 21/100,000 per annum (approximately 25,000 p.a. new cases), mostly affecting males [31].The mortality rate is very high, approximately 16,500 deaths annually (15/100,000 p.a.), about half of which are from cancers of the oropharynx (~ 8500 p.a.) [31]. A survey by WHO in 2004 estimated that ~130,000 head and neck cancer patients existed in Bangladesh [32]. Although tobacco (smokeless or smoked), frequently together with areca nut, is the major risk factor, a recent study gives a reduction of tobacco use from 42.4% to 36.3% between years 2009 and 2012, [33] among those aged 15 and above; in the 40–54 year age group, this fell from 64% to 54%. Bangladesh is a rapidly “progressing” country, embracing much of the good and bad characteristics of western culture. This is associated with reductions in the use of smokeless tobacco and areca products, and increasing smoking rates. There is no published evidence to assess changes in sexual behaviours, and it has become necessary to investigate the true prevalence of HPV-associated HNSCC in the country and the wider region.

The reported prevalence of HPV-associated HNSCC varies widely across the world [34]. Possible reasons for this include geographical location, differences in sexual behaviours and other lifestyle factors, inclusion of mixed ethnicities and, importantly, differences in laboratory detection methods. Sensitivities of the latter are critical, so that lesions with low copy numbers of HPV may fail to be correctly ascribed. It is necessary to use a highly sensitive but controlled detection system, a well-characterised study population and site-specific tissue samples for accurate estimation of HPV prevalence in HNSCC. Although PCR-based assays, in situ hybridization (ISH) and p16 IHC are widely available, there is no consensus on the optimum technique for routine screening. Recent studies have largely used PCR, which is sensitive and cost effective [34, 35]. However, these can be too sensitive and may amplify contaminant HPV from the laboratory environment if appropriate measures are not taken. With PCR, most laboratories use the L1 gene of HPV as the amplification target, as it is more stable in fixed tissues and its product is equivalent to whole HPV genome. It is important to realise that whatever gene is chosen for amplification, be it L1 or the E6/E7 oncogenes, positive results merely indicate the presence of the virus: they show association, not causation. Thus targeting E6/E7 mRNA is, conceptually, ideal as these are the driver genes for oncogenesis, but this is only possible if the tissue is fresh as such RNA degrades quickly over time. ISH offers specificity, particular in the sense of being able to show nuclear location, and thus presumptive viral integration, but this is time consuming, often has background staining and is less sensitive [36].

We applied a combination of nested PCR- for detection of HPV DNA and IHC for expression of p16 [37]. Combination of two sets of primers in nested PCR has been shown in previous studies to be efficient and accurate with oro-pharyngeal tissue [38–40]. The p16 protein has recently emerged as an important biomarker for HPV in HNSCC. In healthy cells, pRb (a cell cycle check point protein) normally supresses the transcription of p16 protein. However, in HPV-related cancers, pRb protein is functionally inactivated by HPV E7 protein, leading to overexpression of p16 [41, 42]. While consensus PCR is highly sensitive, without p16 IHC, the clinical relevance of HPV infection might be falsely interpreted as the presence of HPV DNA does not necessarily indicate that the virus is biologically active in the tumour [36]. On the other hand, simplicity, cost effectiveness and high sensitivity make p16 IHC attractive as a surrogate marker, even in the absence of a direct mechanistic association between HPV integration and p16 overexpression. Moreover, SCC of the oral cavity and of the larynx typically bear low numbers of transcriptionally active HPVs, suggesting that the expression of p16 at these sites may be elevated via a non-viral mechanism, leading to a false positive interpretation [43]. In spite of this, the specificity of p16, and its acceptable sensitivity, makes it a valuable tool. If there were no cost limitations, both methods would be used.

A recent study from Australia showed greater discordance than we report here between the detection of HPV DNA in HNSCC tissue samples [50/248 (20%)] and p16 IHC [61/248 (28%)] [44]. A study from the USA was discordant in the opposite direction, with 54/79 (61%) of cases HPV DNA positive by PCR but only 19/79 (24%) p16 positive [45]. The reasons for these discordances could be either the high sensitivity of nested PCR or the low sensitivity of p16 IHC (especially in the oral cavity and larynx) due to somatic alterations in chromosome 9 [46]. Moreover, subjective evaluation/lack of standardised p16 scoring criteria make comparison of different studies dangerous. Although a high cut-off point for p16 staining (> 50% tumour cells moderately or strongly stained) has been used for cervical cancer, a low cut-off point, used for cases with limited p16 staining, has the potential for over-diagnosing the involvement of HPV [47]. It is not clearly understood whether HPV DNA positive cancers with limited p16 positivity are HPV-driven or whether the p16 is silenced by mutations or by DNA methylation of promoter regions, as has been reported in cervical cancers [48].

The overall worldwide prevalence of HPV in HNSCC averages approximately 30% with wide variation depending on geographical location and tumour sites [34]. Few studies from South Asia have been published; the majority of them from India, and these suggest an overall prevalence of approximately 37% [11]. We found 21% of total HNSCC cases positive for HPV DNA in this Bangladeshi series, highest in the oropharynx (~ 37%), lowest in the larynx (~ 12.5 %). High prevalence in oral cavity SCC has been reported from South East Asia (~48%), Eastern Asia (~43%) and South Asia (~38%) [11]; whereas our study shows slightly lower HPV frequencies in OCSCC (~20%). In oropharyngeal cancer, our study shows the prevalence of HPV to be higher compared to a recent Indian study, where the proportion was approximately 23% [49].Our findings for laryngeal cancer (~ 12.5%) are lower than the overall HPV-positive laryngeal cancer prevalence so far reported in the Asia-Pacific region (23.6%) [11] .

To our knowledge, there is only one published study from Bangladesh describing the prevalence of HPV in HNSCC, using conventional PCR methods. This had a small sample size (*n* = 34), included samples only from the oral cavity and found just 3% of HPV positive cases [50]. Possible reasons for this discordance could be a less sensitive detection method or samples having poor DNA quality: it was also published several years ago, and the prevalence may well be rising nowadays in Bangladesh, as elsewhere in the world.

HPV16 is the most commonly detected HPV type, being present in 90% of cases of HPV-positive HNSCCs worldwide [51]. Our data are similar: 33 out of 36 (~92%) were HPV16 and all were single HPV infections. Similar findings are reported from North America, Europe and the Asia-Pacific [11, 34]. This is likely to be related to social norms, as a relatively high proportion of men in western countries tend to have multiple sexual – including the practice of oral sex – partners [52]. Our data show the prevalence of HPV to be inversely correlated with age, a significantly higher prevalence being seen in those less than 60 yrs. old (mean age 54.2), compared with older patients (*p* = 0.025). This accords with the mean age reported from Australia (55.2 years), North America (58 years) and Europe (< 60 years), although the mean age of our Bangladeshi HPV-positive HNSCC patients was slightly higher than reported from other South Asian countries (52.8 years), especially India [6, 11].

In our study, HPV-positive cases tended to have high expression of p16, an association which was statistically significant: however, our HPV-positive cases had low to moderate expression of Cyclin D1, pRb and p53 proteins, none of which were statistically significant. The negative correlation between HPV status and Cyclin D & pRb perhaps indicates that malignancy may not need both aberrant Cyclin D1 and pRb pathways. Our findings in this respect match previously reported studies from China, India and Australia [44, 53, 54].

Several recent studies have demonstrated the prognostic value of cell cycle markers in HPV-positive HNSCC patients. A recent cohort study suggests that high p16 expression is correlated with better survival: p16 positive and HPV-positive cases had a 2 year disease free survival of 86.2%, (95% CI 79–91.1) compared to p16 negative and HPV–negative cases of only 44.2%, (CI 30.2–58.1) [55]. Some studies have suggested that high expressions of p53, Cyclin D1 or pRb also relate to poor prognosis. For example a study from Sweden suggests that patients with HPV + ve DNA and low/absent p53 had a 5-year survival of 88.2% compared to 33.3% for HPV + ve cases with high p53 expression. Patients with HPV-negative and low/absent p53 had a better survival than HPV-negative but high p53 cases (52.6% vs 9.1%) [56].

A comprehensive study of 226 patients from Australia suggests a strong association between HPV positivity and downregulation or absent expression of Cyclin D1.In an HPV-positive tumour group, Cyclin D1-positive cancers had 8 fold-increased risk of poor prognosis compared to Cyclin D1-negative cancers with 3.3 years overall survival. However, the effect of Cyclin D1 was small in HPV-negative HNSCC [57]. A strong inverse relationship between pRb and p16 expressions has also been reported: cases with low pRb and high p16 expression had better survival [58].

There are several limitations to the present study. We have a modest cohort size, with small numbers in some anatomical subsites. Use of FFPE archival samples resulted in poor quality of DNA in some samples, which had to be excluded. Nevertheless, most amplified the L1 target satisfactorily. Expression of E6/E7 mRNA could not be explored in FFPE tissues, as fresh or frozen tissues were not available. We also have limited information on tobacco, areca nut and alcohol habits, and on the sexual lives of our cases. Most importantly no treatment and patient outcome information was available to us.

This is the first comprehensive study from Bangladesh and one of the first studies from South Asia to use a combination of detection methods for HPV and their interrelationship with cell cycle markers of putative prognostic value. Nevertheless our cases were derived from a single pathology laboratory, which may limit generalizability. However, this is the largest public laboratory in the nation and receives a wide range of patients and tissue samples from all corners of Bangladesh.

## Conclusion

Our data show that HPV is associated with, and probably responsible for ~21% of HNSCC in Bangladesh. Because it is now well known that such cases respond comparatively well to treatment, routine assessment of HPV status in HNSCC should be mandated. We strongly recommend the use of ICD-10 for proper site-specific classification of cases. We urge the Bangladesh Government to mandate Tumour Boards and perform longitudinal studies on HNC to confirm the importance of HPV in treatment planning. We recommend the use of both p16 IHC and PCR-based detection of virus, though p16 alone is a useful surrogate marker. Routine use of IHC for the status of p53, pRb and cyclin D1, does not seem to be indicated.

## Abbreviations

FFPE: Formalin fixed paraffin embedded
HNC: Head and neck cancer
HNSCC: Head and neck squamous cell carcinoma
HPV: Human papillomavirus
IARC: International Agency for Research on Cancer
ICD −10: International Classification of Disease Version 10
IHC: Immunohistochemistry
OCSCC: Oral cavity squamous cell carcinoma
OPSCC: Oropharyngeal squamous cell carcinoma
PCR: Polymerase chain reaction
